# Henna-induced Hemolysis and Acute Kidney Injury in an 85-year-old Man; a Case Report 

**Published:** 2020-10-14

**Authors:** Sahel Asgari, Mohsen Esfandbod, Maryam Haghshomar

**Affiliations:** 1 Faculty of Medicine, Tehran University of Medical Sciences, Tehran, Iran. Email: sahelasgari@yahoo.com.; 2 Hematology-Oncology and Stem Cell Transplantation Research Center, Tehran University of Medical Sciences, Tehran, Iran. Email: mohsen.esfandbod@gmail.com.; 3 Faculty of Medicine, Students' Scientific Research Center, Tehran University of Medical Sciences, Tehran, Iran. Email: Maryam_haghshomar@yahoo.com.

**Keywords:** Case reports, hemolysis, Lawsonia plant, toxicity, herbal medicine, anemia, hemolytic, acute kidney injury

## Abstract

Henna is a commonly used traditional cosmetic agent, which also holds medical potentials and is used to treat skin lesions including seborrheic dermatitis or fungal infections and also has possible anti-inflammatory effects. It contains lawsone (2-hydroxy-1,4-naphthoquinone) and, therefore, has the potential to induce oxidative hemolysis. Henna-induced hemolysis has been previously reported in children with Glucose 6-Phosphate Dehydrogenase Deficiency. Here, we report an 85-year-old man who developed hemolytic anemia and acute kidney injury following oral consumption of henna to help his dyspnea. He was treated with hydration, bicarbonate, and dexamethasone. Over the course of hospitalization, the patient developed ventilator-associated pneumonia and was treated with antibiotic. He was discharged after one month. This finding is of high importance due to common use of henna, especially among people with false beliefs regarding traditional and herbal medicine, and highlights the role of a full history taking.

## Introduction

Hemolytic anemia is a situation in which disruption of red blood cells (RBCs) happens with a faster speed than their reproduction and is accompanied with a decrease in hemoglobin. Its etiology is divided into inherited and acquired. Certain medications, chemicals, toxins, and herbal medicines can cause hemolytic anemia (1). Drug-induced immune hemolytic anemia (DIIHA) is an uncommon phenomenon caused by an immune response to a drug following its administration (2). This reaction is caused either by antibody-mediated complement activation, which results in an intravascular hemolysis, or antibody-mediated phagocytosis, which results in an extravascular hemolysis. Since the first discovery of DIIHA, a large number of drugs have been reported to cause positive Direct Anti-globulin Test (DAT) (3). Penicillin and its derivates, cephalosporins, β-lactamase inhibitors, and quinidine are the most common causes of this uncommon complication (4). Chemicals like phenylhydrazine-HCL can induce hemolytic anemia (5). This phenomenon is most likely a side effect of oxidative stress. Henna has been reported to lead to this problem in previous case reports (6, 7). Here, we report an 85-year-old man who developed hemolytic anemia and acute kidney injury following oral consumption of henna to help his dyspnea. Recognizing this rare complication caused by a common traditional cosmetic agent is of high importance due to common use of henna, especially among people with false beliefs regarding traditional and herbal medicine, and highlights the role of a full history taking.

## Case presentation:

85-year-old men presented to emergency department (ED) with chief complain of abdominal pain and constipation from 3 days prior to admission. He hadn’t passed any gas during those days. Pain was not related to eating. On admission, his general examination and vital signs showed restlessness, icteric sclera, generalized wheezing in lung auscultation, peri-umbilical tenderness, mild tachycardia (heart rate 100 bpm), tachypnea (respiratory rate 32), decreased O_2_ saturation (78% in room air) and wide pulse pressure (150/70 mmHg). The patient was admitted to general surgery service in ED. Abdominal ultrasonography and computed tomography (CT) scan were normal and chest X-ray showed some levels of increased air. Medical history of the patient revealed nothing significant, except for some unclear respiratory system problems, dyspnea suggestive of possible smoking history, and chronic obstructive pulmonary disease (COPD). 

Due to presence of jaundice, an initial lab examination was performed with the aim of evaluating liver and gallbladder function, which found reduced hemoglobin (HB) (9.5 g/dL) and RBC count (3000000 number/ml) (Table 1). Moreover, lab results showed increase in Lactate dehydrogenase (LDH)(3273 U/L), C-Reactive Protein (CRP) (139.6 mg/L), Ferritin (>2000 μg/L), Aspartate aminotransferase (AST U/L) (78), prothrombin time (PT) (14.7 seconds), international normalized ratio (INR) (1.17 seconds), potassium (K) (5.93 mmol/L), urea (94 mg/dL), white blood cell (WBC) (36000 number/ml), bilirubin total (9.93 mg/dL), and bilirubin direct (1.02 mg/dL) (Table 1). Urine analysis (UA), manifested a 4+ proteinuria and 1+ glycosuria (Table 2). Morphine was present in urine toxicology (Table 2). 

 On the third day of admission, lab tests were ordered following consultation with an internal medicine specialist due to patient’s fever and loss of consciousness. On that day, the patient arrested but fortunately successful cardiopulmonary resuscitation (CPR) was performed. Emergency service intubated the patient and started full cardiac monitoring. Lab results showed hemolysis with a severe loss of RBC count (1030000 number/ml) and HB level (3.5 g/dL) along with sustained increased ferritin (>2000 μg/L), urea(130), bilirubin total (6.68 mg/dL), and bilirubin direct (1.8 mg/dL). Furthermore, direct and indirect Coombs test appeared negative and Glucose 6 Phosphate Dehydrogenase (G6PD) levels were in normal range (12.0 units/g). Moreover, the patient had developed acute kidney injury (AKI) marked by an elevated creatinine (2.23 mg/dL).

Peripheral blood smear showed schistocytes and other fragmented RBCs. Elevated serum bilirubin, alanine aminotransferase (ALT), lactate dehydrogenase (LDH), and peripheral blood smear results led us to hemolytic anemia diagnosis. 

Following the diagnosis of hemolytic anemia, the patient’s diet was reinvestigated through his family members and his wife, which revealed oral consumption of henna. Henna was prescribed by a traditional medicine physician to help the patient’s dyspnea. Henna powder was dissolved in a glass of water and the patient had drank a glass a day, for 2 consecutive days prior to admission. Hematology consultant made a DIIHA diagnosis based on the history of Henna consumption, clinical findings, and laboratory results of hemolysis. Following this diagnosis, 4 mg dexamethasone three times a day (TDS) was prescribed for the patient. Hydration and bicarbonate therapy were performed to treat the AKI. 

The patient’s condition was improved; however, after a few days he developed fever and a ventilator-associated pneumonia (VAP) was diagnosed, and an infectious disease specialist started antibiotic therapy. The patient was discharged after one month. 

Two weeks later, the patient referred to the hospital with CBC, Cr level, and G6PD level results. G6PD level was rechecked as the enzyme levels might not be low in the acute hemolysis phase. All test appeared within normal range. 

## Discussion

Henna is a traditional cosmetic agent applied over skin, hair, or nails as a dye. It is derived from Lawsonia Alba shrub and it contains Lawsone (2hydroxy–1,4 napthoquinon). Black henna contains Paraphenylenediamine (PPD). In some countries in Africa, south east Asia, and middle east, henna is commonly used in ceremonies to create patterns on the skin. In addition to cosmetic uses, it has anti-inflammatory affects, which makes it suitable for seborrheic dermatitis or fungal infections therapy. Studies have suggested anti-inflammatory, antipyretic, and analgesic effects for henna (8). 

Lawsone has a structure and redox potential similar to ortho-substituted 1, 4-naphthoquinones, which is an oxidant of G6PD and normal RBCs, and henna absorption may lead to oxidative injury (9). PPD is also an oxidative chemical allergen that can cause adverse systemic effects such as laryngeal edema and respiratory distress, often demanding emergency tracheostomy and leading to AKI, rhabdomyolysis, and multiple organ failure (10). The patient had obstruction symptoms like abdominal pain and constipation, and also suffered from AKI and acute respiratory distress, which led to his intubation, and taken together, they seem highly compatible with henna toxicity.

Although henna absorption has a high risk of hemolytic anemia, merely a few reports of DIIHA due to henna application are present. Cases report acute renal failure associated with henna use along with hemolytic anemia. Previously, most cases of henna-induced hemolysis had been reported in newborns and infants. Deveciglu et al. reported hemolytic anemia and acute renal failure after cutaneous application of henna to abdomen, intertriginous region, and legs for treating diaper rash, developed in a 27-day-old boy (6). Seyedzadeh et al. found severe acute hemolysis in a 42-day old infant followed by topical application of henna for treating his napkin dermatitis (11). Ilkhanipur et al. described a 35-day old, G6PD-deficient boy who had advanced jaundice, hemogluinuria, and kernicterus symptoms after cutaneous intake of henna (12). 

Cases with older age, are also present. Through investigating venous blood exposure with different levels of lawsone in 15 healthy and 4 G6PD-deficient adults, Zinkham et al. found that henna is capable of causing oxidative hemolysis, which is more severe and more probable in G6PD-deficient patients (8). Kheir et al. reported a case of life-threatening henna-induced hemolytic anemia after application of henna to skin in Sudan. This case was a 6-year-old boy who was diagnosed with G6PD deficiency after workup for hemolysis (13). Raupp et al. found 4 cases of hemolytic crisis following topical application of henna, all diagnosed with G6PD deficiency. One male and one female neonate, and two boys aged three and 4 years made up the cases (7). Qurashi et al. reported a young Saudi male who developed AKI and intravascular hemolysis following ingestion of henna mixed with para-phenylenediamine (14). 

Moreover, a case of severe hemolytic anemia was reported after voluntary ingestion of henna to induce abortion. The 17-year-old girl year was later diagnosed with G6PD deficiency (15).

We are the first to report henna-induced hemolytic anemia in a non-G6PD-deficient patient after oral consumption of henna at an old age. It can be assumed that oral intake of henna will be more potent in causing hemolysis compared to cutaneous absorption.

**Table 1:** Laboratory findings of the patient

**Measures**	**Day 1**	**Day 3 **	**Normal Range**
pH	7.40	-	7.35-7.45
PCO2 (torr)	38.7	-	35-45
Lactate dehydrogenase (U/L)	3273	5010	<480
C-reactive protein (mg/L)	139.6	-	<3.0
Ferritin (μg/L)	>2000	>2000	35-300
Folic Acid (ng/mL)	13.3	-	2-20
Vitamin B12 (ng/mL)	548	-	138-652
Blood Sugar (mmol/L)	200	180	<200
Urea (mg/dL)	94	130	18-55
Creatinine (mg/dL)	1.13	2.23	0.6-1.2
Natrium (mmol/L)	135	149.8	135-145
Potassium (mmol/L)	5.93	5.12	3.5-5
Calcium (mmol/L)	-	8.3	8.2-10.3
Magnesium (mmol/L)	-	2.7	1.8-2.6
Aspartate Aminotransferase (U/L)	78	-	10-40
Alanine Aminotransferase (U/L)	52	-	7-56
Alkaline Phosphatase (U/L)	293	-	44-147
Bilirubin Total (mg/dL)	9.93	6.68	0.1-1.2
Bilirubin Direct (mg/dL)	1.02	1.8	<0.4
Prothrombin Time (seconds)	14.7	-	11-13.5
Partial Thromboplastin Time (seconds)	31	-	30-40
International Normalized Ratio (seconds)	1.17	-	<1.1
Amylase (U/L)	45	-	30-110
White Blood Cell (number/mm³)	36000	39900	4500-11000
Red Blood Cell, million (number/mm³)	3.0	1.03	4.32-5.72
Hemoglobin (g/dL)	9.5	3.5	13.5-17.5
Hematocrit (%)	-	11	41-52
Mean corpuscular volume (femtoliters)	95.72	106.8	80-96
MCH* (picograms/cell)	31.25	33.98	27-33
MCHC** (g/dL)	32.65	31.28	33-36
Platelets (mm³)	285000	207000	150000-450000
Red cell distribution (width %)	18.8	23	11.5-14.5
Mean platelet volume (femtoliters)	-	11.3	8-12
Platelet Distribution (width %)	-	15.5	10.0-17.9
Neutrophil (%)	-	70.7	40-60
Lymphocyte (%)	-	27.7	20-40
Mixed (%)	-	1.6	4-8

*: Mean corpuscular hemoglobin; **: mean corpuscular hemoglobin concentration.

**Table 2:** Urine analysis and urine toxicology findings of the patient

**Urine Toxicology**	**Result**	**Urine Analysis**	**Result**
**Nitrite **	Negative	**pH**	6.5
**Morphine**	Positive	**Specific Gravity**	1.01
**Amphetamine**	Negative	**Color**	Yellow
**Benzodiazepines**	Negative	**White Blood Cell**	4-5
**Buprenorphine**	Negative	**Red Blood Cell**	0-1
**Cocaine**	Negative	**Glucose**	1+
**Methadone**	Negative	**Protein**	4+
**Tricyclic Antidepressant**	Negative	**-**	**-**

## Conclusion:

In conclusion, henna-induced hemolytic anemia is rare and most cases occur in G6PD-deficient individuals during their first months of life or at a young age; however, the chance of hemolysis occurrence in healthy or old individuals cannot be ruled out. Since henna is a commonly used herbal agent, clinical suspicion toward henna-induced hemolysis after unexplained hyperbilirubinemia in patients with a history of henna intake is of high importance. More observations and further investigation into the mechanism of this hemolytic reaction seems necessary. It should be highlighted that obtaining a full history of herbal medicines remains critical due to patients’ false beliefs.

***List of abbreviations***

Drug-induced immune hemolytic anemia (DIIHA); red blood cells (RBC); Direct Antiglobulin Test (DAT); emergency room (ER); hemoglobin (HB); Lactate dehydrogenase (LDH); C-Reactive Protein (CRP), Ferritin; Aspartate aminotransferase (AST); prothrombin time (PT); international normalized ratio (INR); potassium (K); white blood cell (WBC); bilirubin total (bili. T); bilirubin direct (bili. D.); Urine analysis (UA); cardiopulmonary resuscitation (CPR); glucose 6 Phosphate Dehydrogenase (G6PD); acute kidney injury (AKI); three times a day (TDS); Ventilator-associated pneumonia (VAP); Paraphenylenediamine (PPD)

## Ethics consideration

This study was approved by department of medical ethics, Tehran University of Medical Sciences with ethic number IR.TUMS.SINAHOSPITAL.REC.1399.045.

## Availability of data and materials

The datasets used during the current study are available from the corresponding author on reasonable request.

## Conflict of interests

The authors declare that they have no competing interests

## Funding

This research was supported by the Tehran University of Medical Sciences.

## Authors' contributions

SA performed the initial examination of the patient and contributed in the final diagnosis and was a contributor in writing the manuscript. ME analyzed and interpreted the patient data regarding the hematological disease. MH was a major contributor in writing the manuscript. All authors read and approved the final manuscript.
